# Abiotic Stress Responses in *Raphanus sativus* L.: Insights into the Role of Endopolyploidization in Stress Adaptation

**DOI:** 10.3390/plants15152320

**Published:** 2026-07-28

**Authors:** Marianna Janová, Michaela Bačovčinová, Vladislav Kolarčik, Renáta Švubová, Pavol Mártonfi

**Affiliations:** 1 Institute of Biology and Ecology, Faculty of Science, Pavol Jozef Šafárik University in Košice, Mánesova 23, SK-040 01 Košice, Slovakiavladislav.kolarcik@upjs.sk (V.K.); pavol.martonfi@upjs.sk (P.M.); 2 Department of Plant Physiology, Faculty of Natural Sciences, Comenius University Bratislava, Ilkovičova 6, Mlynská dolina, SK-842 15 Bratislava, Slovakia; 3 Botanical Garden, Pavol Jozef Šafárik University in Košice, Mánesova 23, SK-040 01 Košice, Slovakia

**Keywords:** abiotic stress, cadmium, endopolyploidy, oxidative stress, *Raphanus sativus*, UV-B

## Abstract

Endoreduplication is a process in which cells repeatedly undergo the S and G phases of the cell cycle without dividing, thereby increasing their ploidy level. Cells with different ploidy levels subsequently form tissues and organs, resulting in a condition called endopolyploidy. This phenomenon plays an important role in plant growth, development, and homeostasis and may also help compensate for adverse environmental conditions, representing one of the mechanisms of stress adaptation. Two radish cultivars, ‘Lada’ and ‘Ester’, were grown hydroponically and treated with 10 or 20 μM cadmium and UV-B irradiation. The level of endopolyploidization and its role in adaptation to abiotic stress were studied by flow cytometry. Stress intensity was evaluated by measuring fresh weight, assimilatory pigment content, and antioxidant enzyme activities. The experimental data confirmed that cadmium exposure represents a critical limiting factor for radish growth, as manifested by a significant reduction in biomass and decreased content of photosynthetic pigments. Induced abiotic stress did not significantly alter endopolyploidization in the petioles and leaves of either cultivar. In the storage hypocotyl of ‘Ester’, the degree of endopolyploidy decreased under 20 μM cadmium treatment, whereas the storage hypocotyl of ‘Lada’ remained relatively stable. The cultivars also differed in their stress response strategies. ‘Ester’ showed higher sensitivity to cadmium stress, accompanied by elevated SOD, GPOX, and APX activity, while ‘Lada’ exhibited higher CAT activity. UV-B irradiation alone caused significant changes only in ‘Ester’, where APX, GPOX, and CAT activities increased. However, the combined effect of cadmium and UV-B was more pronounced, particularly in the chlorophyll *a*/*b* ratio and antioxidant enzyme activities in both cultivars. The results indicate that UV-B acts more as a modulator of the physiological response to cadmium than as a primary stress factor. These findings suggest that the maintenance of endopolyploidy may be associated with greater tolerance to the combined action of cadmium and UV-B stress.

## 1. Introduction

Endoreduplication is one of the alternatives to the mitotic cell cycle, and in plants it is the primary mechanism leading to endopolyploidy—the occurrence of cells with different ploidy levels within an organism [1,2,3]. Endoreduplication is tightly regulated at the molecular and phytohormonal level, similarly to the canonical cell cycle. Cell-cycle progression is regulated by two families of cyclin-dependent kinases (CDKs)—CDKA and CDKB [4]. Their regulation is carried out by either phosphorylation, cyclin-dependent kinase inhibitors (CKIs) or cyclins (CYCs), which bind to them and form complexes [3]. The most important complexes regulating cell-cycle progression are the CDK–CYCD and CDK–CYCA complexes, which regulate the cell cycle at checkpoints in the transition between G1 and S and between S and M phases, respectively [5]. Their regulation keeps the cell arrested in the S and G phases of the cell cycle, while DNA replication occurs without the genetic material being divided between daughter cells, thereby increasing the ploidy level [6]. Endoreduplication is terminated by the negative regulators Maturation-Promoting Factor (MPF) and CYCA2;3, which are responsible for controlling DNA replication, thus maintaining genome stability [6].

At the level of phytohormonal regulation, auxins (AUXs) and cytokinins (CKs) are directly involved in the control of endoreduplication, and the endopolyploidy level is a result of the balance between activities of these two phytohormones [7,8]. Other phytohormones also influence the ploidy level, but they probably affect endopolyploidization through the regulation of AUX and CK biosynthesis and signalling [8]. The increase in the ploidy level of cells is indirectly caused by gibberellins and ethylene [6,9]. In contrast, brassinosteroids [10,11], abscisic acid [6], salicylic acid [12] and jasmonic acid [13] often exert a negative effect by shifting the balance between AUXs and CKs, resulting in decreased endopolyploidy levels by inducing cell division or keeping the cell arrested in the G2 phase [8,14,15].

Endopolyploidization occurs within several evolutionary branches of plants. It is present in red, brown and green algae [16,17,18] and occurs only rarely in ferns and gymnosperms [19]. By contrast, it is abundantly represented in mosses [20,21,22] and angiosperms, with angiosperms being most thoroughly studied [2,14,19]. Based on knowledge about the occurrence of endopolyploidy, it appears that endopolyploidy arose independently in several evolutionary branches [19]. The most significant factor that influences the occurrence and variability of endopolyploidy is the position of the taxon in phylogeny and belonging to a certain family [2]. Endopolyploidy is a common cytological feature in many important crop families, including Poaceae [23,24], Solanaceae [25,26], and Brassicaceae [27,28,29]. In addition, there is a weak negative correlation with genome size [2,30], as endopolyploidy is more common in species with small genomes due to the difficulty of replicating a large genome [30]. Considerable variability has been observed not only between different species but also between different ecotypes or chemotypes, and between diploids and polyploids of the same species [31].

Endopolyploidy provides many advantages for plants, particularly in relation to growth and development. It promotes cell enlargement and consequently contributes to the growth of the entire organism, as the combination of cell division and cell expansion represents one of the most efficient growth strategies [3]. Additional rounds of DNA replication also support the specific requirements of tissues and organs by supplying material for the formation of cellular structures [15,32]. Increased DNA content encoding proteins and other metabolites may further influence cell specialization [19].

The functional role of endopolyploidy in plants has been demonstrated in examples of specialized plant structures in both model and non-model plants [33,34,35], and new cases of specialized endopolyploid cells are still being discovered, where endopolyploidy is closely linked to their morphological and functional specific roles, e.g., suspensor cells, bulliform cells, or epidermal cells accumulating sodium in some halophytes [36,37,38].

From an economic and commercial perspective, plants with higher levels of endopolyploidization are often more valuable because they produce larger flowers [39,40], larger fruits [3,41], and greater amounts of metabolites [19], or they may exhibit a shorter growth period compared with individuals showing lower levels of endopolyploidization [2]. In addition, this phenomenon contributes to the maintenance of cellular homeostasis and may participate in adaptation to biotic and abiotic stress [15,19]. Stress conditions stimulate the synthesis of specific phytohormones, which can trigger a transition from the mitotic cell cycle to endoreduplication [15]. Enhanced ploidy levels are subsequently associated with increased production of secondary metabolites and greater support for the formation and growth of specialized structures such as root hairs and trichomes [3,32]. When DNA damage occurs during stress exposure, cells may partially compensate through endoreduplication [42], for example by increasing the copy number of genes encoding protective enzymes such as photolyases [43].

Cadmium is a heavy metal that pollutes soil and water in connection with geological and anthropogenic activities [44]. Plants, depending on the species, can tolerate low concentrations of cadmium. However, higher cadmium concentrations have a negative impact on photosynthesis and respiration, growth and development of tissues, and the ability of the plant to absorb water and minerals from the soil [44]. The mechanisms by which cadmium affects endopolyploidy remain unclear. Several studies indicate that cadmium can increase endopolyploidy, especially in trichomes [44] or in roots [45,46]. In contrast, in leaves, it can decrease the number of endocycles, probably due to post-translational alterations of CYCs and CDKs involved in the progression of either the mitotic cycle or the endoreduplication cycle [44].

UV-B radiation is part of the light spectrum with wavelengths of 290–315 nm [42] and negatively affects plant growth. Excessive UV-B exposure causes DNA damage by the formation of cyclobutane–pyrimidine dimers (CPDs), which inhibit transcription and translation [43]. In addition, it negatively affects plant growth and leaf photosynthetic activity [47]. The transition to the endoreduplication cycle can be induced by the UV-B photoreceptor UVR8 or by inhibiting the function of DEL1 proteins [42,48].

Both cadmium and UV-B cause a reduction in the synthesis of assimilatory pigments and the accumulation of reactive oxygen species (ROS), which rapidly reduce the overall fitness of the plant and can later lead to cell and plant death [49]. In response to oxidative stress, the synthesis of enzymatic and non-enzymatic antioxidants that detoxify ROS in the plant is triggered [49].

This study aimed to investigate the effects of cadmium, UV-B radiation, and their combined treatment on endopolyploidization and oxidative stress responses in two cultivars of *Raphanus sativus* L., with particular emphasis on the potential role of endopolyploidy in adaptation to abiotic stress. *Raphanus sativus* is an agronomically important root vegetable cultivated worldwide for its edible storage hypocotyl. During hypocotyl development, this organ undergoes extensive endoreduplication, making radish a suitable model for investigating the relationship between endopolyploidy and growth under abiotic stress conditions [27]. Despite its agricultural importance and documented high endopolyploidy levels, the effects of combined cadmium and UV-B stress on endopolyploidy and antioxidant responses in radish have not been investigated to date.

## 2. Materials and Methods

### 2.1. Plant Material

Seeds of two cultivars of *Raphanus sativus*, ‘Ester’ and ‘Lada’, were obtained from SemenaOnline, s.r.o (Jeneč, Czech Republic; available at osiva-semena.sk). ‘Lada’ is a mid–early cultivar suitable for year-round cultivation with a vegetation period of 33–37 days. ‘Ester’ is an early radish cultivar, also suitable for year-round cultivation with the vegetation period slightly shorter (31–34 days). The seeds were pre-germinated on perlite in Petri dishes for 5 days and then grown in hydroponics. After 5 days, the plants were transferred to 700 mL pots, where they were grown in modified Hoagland solution (0.10 mmol/L KH_2_PO_4_, 1.25 mmol/L KNO_3_, 1.80 mmol/L Ca(NO_3_)_2_·4H_2_O, 0.20 mmol/L MgSO_4_·7H_2_O, and 0.20 mmol/L Fe-EDTA; microelements: 11.56 μmol/L H_3_BO_3_, 2.29 μmol/L MnCl_2_·4H_2_O, 0.191 μmol/L ZnSO_4_·7H_2_O, 0.08 μmol/L CuSO_4_ · 5H_2_O, and 0.018 μmol/L (NH_4_)_6_Mo_7_O_24_·4H_2_O) at a temperature of 21 ± 2 °C with a 16 h light/8 h dark photoperiod.

The experiment was arranged in four independent experimental blocks, each containing all six treatment combinations (0, 10, and 20 μM CdCl_2_, with and without UV-B irradiation). Each treatment was represented by one pot per block, with two plants grown in each pot, resulting in a total of 48 plants analysed per cultivar. The pots were arranged in a fixed 4 × 6 grid for each treatment throughout the experiment, and their spatial position (row and column) was recorded for subsequent statistical analysis to account for potential positional effects within the growth area. After 14 days, the plants were exposed to cadmium stress by adding CdCl_2_ to the medium to obtain 10 and 20 μM concentrations. Hoagland medium was changed weekly. The plants were exposed to UV-B radiation by irradiating with a UV-B fluorescent lamp G8T5E (Sayko Denki, Tokyo, Japan; 0.68 mW/cm^2^) for 2 h on days 30, 35 and 42. The UV-B regime was selected to represent a moderate UV-B treatment sufficient to activate stress-response pathways without causing severe photodamage (Supplementary Figure S1) [50]. Fifty-day-old plants were used for analysis of endopolyploidy, and the rest of the material was frozen in liquid nitrogen and stored at −80 °C.

### 2.2. Measurement of Plant Biomass

Fifty-day-old plants were removed from pots and dried of excess Hoagland medium. Fresh weights (FWs) of roots (including the storage hypocotyl) and shoots were weighed separately for the analysis of stress-induced growth changes.

### 2.3. Flow Cytometric Analysis of Endopolyploidy

Samples were prepared from fresh storage hypocotyls, petioles and leaf blades of the largest leaf (usually the third/fourth leaf) of the rosette. For sample preparation, a ~1 cm^2^ disc without large veins excised from the middle part of the leaf blade, a ~1 cm long section from the middle part of the leaf petiole and half of a ~1 mm thick (in vertical direction) section of the middle part of the hypocotyl tuber were used. The tissues were homogenized by cutting in GPB buffer (0.5 mmol/L spermine × 4HCl, 30 mmol/L sodium citrate, 20 mmol/L MOPS, 80 mmol/L KCl, 20 mmol/L NaCl, and 0.5% [*v*/*v*] Triton X-100, pH 7.0) [51] with a razor blade to release the nuclei into the solution. The larger parts of tissues were removed by filtering through a 42 μm nylon mesh. After filtration, 30 μL of RNase (1 mg/mL) and 4 μL of β-mercaptoethanol were added to the samples, and DNA was stained with 30 μL of propidium iodide (1 mg/mL). Endopolyploidy was measured using a CyFLow ML cytometer (Partec GmbH, Münster, Germany) equipped with a 532 nm green laser and 590 nm optical filter. On the resulting flow cytometry histograms, we manually marked the peaks and the numbers of nuclei, with individual ploidy levels read in Flomax ver. 2.70 (Partec GmbH, Münster, Germany). Endopolyploidization was expressed as the endoreduplication index (EI), which we calculated according to Barow and Meister [2] using the following formula:

EI=(0×n2C+1×n4C+2×n8C+3×n16C+…)/(n2C+n4C+n8C+n16C+…),

where *n2C*, *n4C*, *n8C*, *n16C*… represents the number of nuclei of the respective ploidy levels.

### 2.4. Concentration of Assimilatory Pigments

Concentration of assimilatory pigments was measured according to Lichtenthaler [52]. A total of 20 mg of leaves were homogenized with MgCO_3_ on a bead homogenizer (TissueLyser II, Qiagen, Hilden, Germany), followed by the addition of 2 mL of chilled 80% acetone. The samples were centrifuged (Centrifuge 5430, Eppendorf, Germany) at 10,000× *g* at 4 °C for 10 min. Absorbances were measured spectrophotometrically on a Jenway 7310 spectrophotometer (Cole-Parmer Ltd. Stone, Staffordshire, UK) at wavelengths of λ = 470, λ = 647, and λ = 663 nm. The concentration of chlorophyll *a*, chlorophyll *b* and carotenoids in mg/g FW was calculated according to the formulas:

$$\mathrm{Chl}\;a=(-12.25\times A_{663}-2.79\times A_{647})$$

$$\mathrm{Chl}\;b=(-21.5\times A_{647}-5.1\times A_{663})$$

$$\mathrm{Car}=-(1000\times A_{470}-1.82\times \mathrm{Chl}\;a-85.05\times \mathrm{Chl}\;b)/198$$

### 2.5. Concentration of Soluble Proteins

Extracts were prepared by homogenizing 150 mg of roots and 250 mg of leaves in liquid nitrogen. Soluble proteins were extracted in extraction buffer (50 mM sodium phosphate buffer, 1 mM EDTA, 2% PVPP, protease inhibitor cOmplete™, and EDTA-free Protease Inhibitor Cocktail). The samples were centrifuged for 15 min at 14,000× *g* at 4 °C. Protein concentration was measured by the Bradford method on a Jenway 7310 spectrophotometer at λ = 595 nm [53]. A calibration curve for calculating the concentration of soluble proteins was prepared using a BSA standard solution (2 mg/mL in distilled water).

### 2.6. Superoxide Dismutase Activity

SOD (E.C.1.15.1.1) activity was determined according to Garcı’a-Limones et al. [54]. The reaction mixture containing 75 μL of protein extract was incubated for 20 min under white, fluorescent light. A reaction mixture without protein extract served as the control. Absorbance was measured on a Jenway 7310 spectrophotometer at a wavelength of λ = 560 nm. The specific activity of SOD was expressed in U/mg of protein according to the formula:

$$Specific\;activity\;of\;SOD=\frac{\left(A_{posit.control}-A_{sample}\right)\times V_{total}}{0.5\times A_{posit.control}\times V_{sample}\times c_{protein}}$$

### 2.7. Catalase Activity

CAT (E.C.1.11.1.6) was determined according to Claiborne [55], with minor modifications. To a mixture of 50 mM sodium phosphate buffer with 3% H_2_O_2_, 50 μL of protein extract was added. The differences in absorbance per minute were measured on a Jenway 6705 UV/Vis spectrophotometer (Bibby Scientific Ltd., Dunmow, UK) at λ = 240 nm. The specific activity in U/mg of protein was calculated according to the formula:

$$Specific\;activity\;of\;CAT=\;\frac{\Delta A_{240}\times V_{total}}{39.4\times t\times V_{sample}\times c_{protein}}$$

### 2.8. Ascorbate Peroxidase Activity

APX (E.C.1.11.1.11) activity was measured according to Nakano and Asada [56]. To the reaction mixture of 50 mM potassium phosphate buffer, 0.5 mM L-ascorbate and 0.1 mM H_2_O_2,_ 50 μL of protein extract was added. The change in absorbance per minute was measured spectrophotometrically on a Jenway 6705 UV/Vis spectrophotometer at λ = 290 nm. The specific activity of APX in U/mg of protein was calculated according to the formula:

$$Specific\;activity\;of\;APX=\;\frac{\Delta A_{290}\times V_{total}}{2.8\times V_{sample}\times c_{protein}}$$

### 2.9. Guaiacol Peroxidase Activity

The activity of GPOX (E.C.1.11.1.7) was determined according to Frič and Fuchs [57]. The change in absorbance per minute was measured at a wavelength of λ = 440 nm on a Jenway 7310 spectrophotometer. The specific activity of GPOX was calculated in U/mg of protein according to the formula [58]:

$$Specific\;activity\;of\;G-POX=\frac{\Delta A_{470}\times V_{total}}{26.6\times V_{sample}\times c_{protein}}$$

### 2.10. Statistical Analysis

All statistical analyses were performed in the R program environment (version 4.5.2) [59]. Prior to hypothesis testing, the normality of residuals was assessed using the Shapiro–Wilk test, and homogeneity of variances was evaluated using Levene’s test. Outliers were identified using the interquartile range (IQR) method. Observations below Q1 − 1.5 × IQR or above Q3 + 1.5 × IQR were considered outliers. In total, 61 observations (6.11% of all measurements) were removed prior to statistical analyses to ensure normality of the data to meet the assumptions required for parametric testing. The identified outliers were distributed across different experimental groups and measured parameters rather than being associated with any particular treatment. They most likely reflected natural biological variability among plants together with occasional technical variation inherent to physiological and biochemical measurements. Their removal did not affect the balance of the experimental design or the overall interpretation of the results, and a sufficient number of replicates were retained for each experimental group (*n* ≥ 3). Differences among studied parameters were evaluated using linear mixed-effects models fitted with the lmer function, from the *lme4* package [60]. Cadmium concentrations, UV-B treatment, and their interaction were defined as fixed effects. Row and column (corresponding to the spatial position of each cultivation pot within the experimental layout), cultivation pot, and individual plant were included as random effects to account for potential spatial variation within the experimental layout, variability among cultivation pots, and biological variability between individual plants. The significance of fixed effects was evaluated using Type II analysis of variance with the Kenward–Roger approximation of degrees of freedom, which is robust to slight imbalances in sample size among groups [61]. Post hoc comparisons of estimated marginal means of individual variants of the experiment were performed by applying the emmeans function from the program *emmeans* package [62]. *p*-values were corrected by Tukey’s method with a significance level of α = 0.05. Figures were generated using the *ggplot2* package [63].

## 3. Results

### 3.1. Plant Biomass

**Figure 1:**
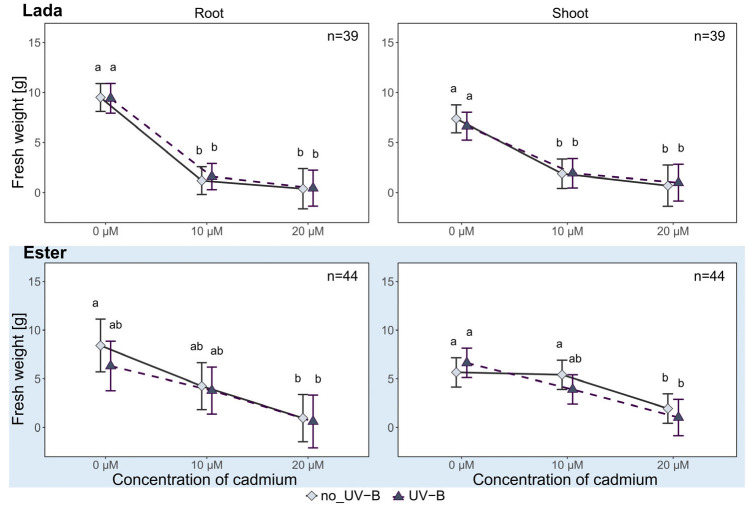
Effect of cadmium, UV-B and their interaction on the fresh weight of roots and shoots in two radish cultivars—‘Lada’ and ‘Ester’. Data points represent means and error bars indicate confidence intervals (CIs); *n* indicates the sample size. Different letters indicate statistically significant differences between treatments (Tukey’s HSD test, *p* < 0.05).

Analysis of plant fresh weight showed that increasing cadmium concentrations negatively affected the growth of both radish cultivars (Figure 1). The inhibitory effect of cadmium was manifested by a significant decrease in the biomass of roots (ANOVA_Cd_: *p* < 0.001) and shoots (ANOVA_Cd_: *p* < 0.001) of the ‘Lada’ cultivar. In ‘Lada’, a sharp decrease in fresh weight occurred at a concentration of just 10 µM Cd. Further increasing the concentration to 20 µM Cd no longer led to statistically significant differences. UV-B at the applied intensity had no significant effect on the roots (ANOVA_UV-B_: *p* > 0.05) or shoots (ANOVA_UV-B_: *p* > 0.05) of ‘Lada’. Likewise, the interaction between cadmium and UV-B was not statistically significant for either roots (ANOVA_Cd×UV-B_: *p* > 0.05) or shoots (ANOVA_Cd×UV-B_: *p* > 0.05), indicating that UV-B did not significantly modify the effect of cadmium on plant biomass.

In the individuals of the cultivar ‘Ester’, we also recorded a significant cadmium-induced decrease in fresh weight in both roots (ANOVA_Cd_: *p* < 0.001) and shoots (ANOVA_Cd_: *p* < 0.001). Roots, which are the first exposed to cadmium, reacted more sensitively than shoots. However, ‘Ester’ showed a higher tolerance to 10 µM Cd than ‘Lada’, as the reduction in biomass was less pronounced. Post hoc analysis revealed that a significant decrease in weight occurred only at 20 µM cadmium. UV-B radiation also showed only negligible differences in this case for roots (ANOVA_UV-B_: *p* > 0.05) and shoots (ANOVA_UV-B_: *p* > 0.05), as did the interaction Cd × UV-B (roots—ANOVA_Cd×UV-B_: *p* > 0.05; shoot—ANOVA_Cd×UV-B_: *p* > 0.05).

### 3.2. Endopolyploidy

**Figure 2:**
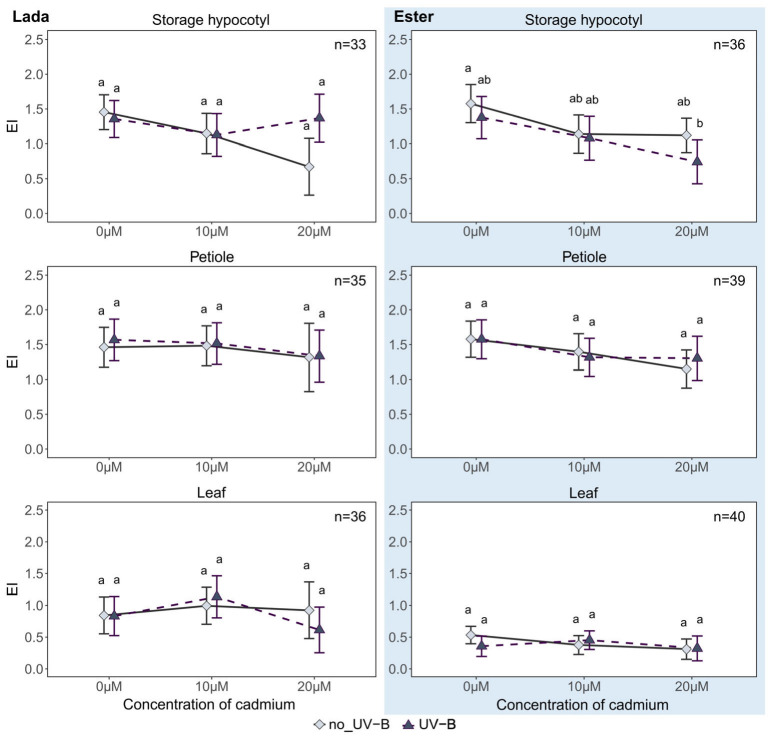
Effect of cadmium, UV-B and their interaction on endopolyploidization of storage hypocotyls, petioles and leaves of two radish cultivars—‘Lada’ and ‘Ester’. Data points represent means and error bars indicate confidence intervals (CIs); *n* indicates the sample size. Different letters indicate statistically significant differences between treatments (Tukey’s HSD test, *p* < 0.05).

Analysis of endopolyploidy by flow cytometry revealed significant differences in EI between organs (Figure 2). In both cultivars, the highest EI values were recorded in the petioles. Conversely, the lowest EI values were calculated for the leaves.

Although there was a slight decrease in EI values in the storage hypocotyls treated with cadmium alone in ‘Lada’, two-way ANOVA did not show statistically significant differences (storage hypocotyls—ANOVA_Cd_: *p* > 0.05; petiole—ANOVA_Cd_: *p* > 0.05; leaf—ANOVA_Cd_: *p* > 0.05). In ‘Lada’, EI did not change significantly in any of the organs even after being treated with UV-B (storage hypocotyls—ANOVA_UV-B_: *p* > 0.05; petiole—ANOVA_UV-B_: *p* > 0.05; leaf—ANOVA_UV-B_: *p* > 0.05) or under the combination of Cd × UV-B treatment (storage hypocotyls—ANOVA_Cd×UV-B_: *p* > 0.05; petiole—ANOVA_Cd×UV-B_: *p* > 0.05; leaf—ANOVA_Cd×UV-B_: *p* > 0.05).

‘Ester’ showed a decrease in endopolyploidization of the storage hypocotyls, with statistically significant differences observed between controls and cadmium-treated plants (ANOVA_Cd_: *p* < 0.05). UV-B (ANOVA_UV-B_: *p* > 0.05) and the combined Cd × UV-B treatment (ANOVA_Cd×UV-B_: *p* > 0.05) did not have a significant effect on the endopolyploidization of the storage hypocotyls. In the petioles, only a slight decrease in EI was indicated, but the differences were not statistically significant for any of the stressors (ANOVA_Cd_: *p* > 0.05; ANOVA_UV-B_: *p* > 0.05; ANOVA_Cd×UV-B_: *p* > 0.05). We recorded a lower level of endopolyploidization in leaves compared to ‘Lada’, with EI values not exceeding 0.6. As in ‘Lada’, endopolyploidization in the leaves was stable under the individual and combined effects of the stressors (ANOVA_Cd_: *p* > 0.05; ANOVA_UV-B_: *p* > 0.05; ANOVA_Cd×UV-B_: *p* > 0.05).

**Figure 3:**
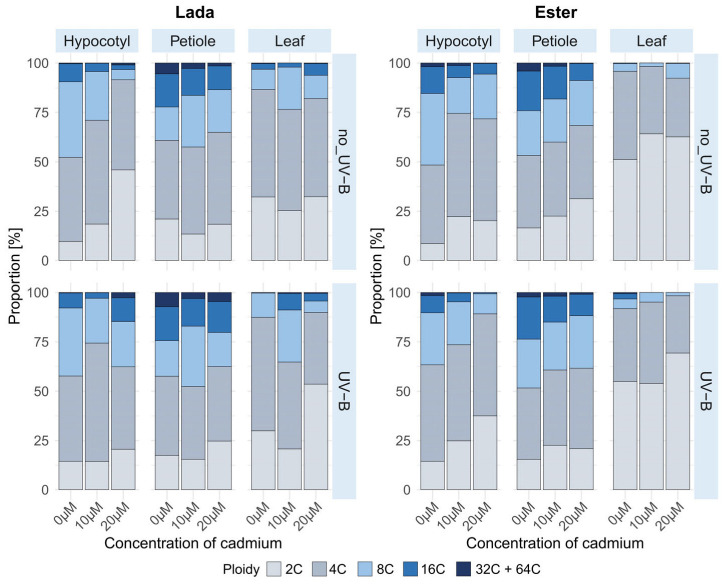
Effect of cadmium, UV-B and their interaction on mean percentual proportions of nuclei with different ploidy levels in storage hypocotyls, petioles and leaves of two radish cultivars—‘Lada’ and ‘Ester’.

The endopolyploidy analysis results were supported by calculating the percentual distribution of nuclei (Figure 3). The proportion of endopolyploid nuclei (4C–8C, particularly 16C–64C) decreased with increasing concentration of cadmium. Storage hypocotyls and petioles showed higher levels of endoreduplication, with a significant proportion of 16C and 32C + 64C nuclei compared to leaves in both radish cultivars.

Cadmium treatment led to a gradual suppression of endoreplication, as evidenced by an increase in the proportion of 2C and 4C nuclei at the expense of higher ploidy levels. The inhibitory effect of abiotic stress was most pronounced in the storage hypocotyls of ‘Ester’. Leaf tissues showed the most stable ploidy distribution, with 2C and 4C nuclei predominating, while their ratio remained preserved even under the influence of the stressors. The differences between the presence and absence of UV-B in the distribution of endopolyploid nuclei were minimal, indicating a dominant effect of cadmium phytotoxicity on cell-cycle progression.

### 3.3. Concentration of Assimilation Pigments

**Figure 4:**
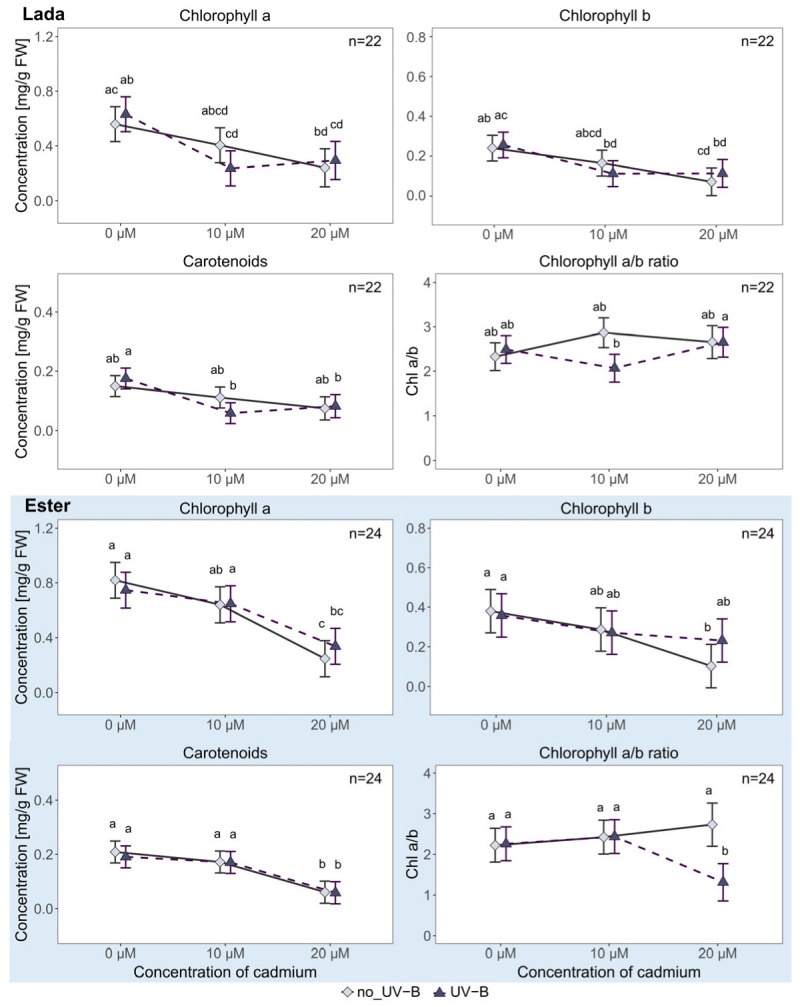
Effect of cadmium, UV-B and their interaction on assimilation pigments concentrations of two radish cultivars—‘Lada’ and ‘Ester’. Data points represent means and error bars indicate confidence intervals (CIs); *n* indicates the sample size. Different letters indicate statistically significant differences between treatments (Tukey’s HSD test, *p* < 0.05).

In both radish cultivars, cadmium and UV-B negatively affected the content of chlorophylls and carotenoids (Figure 4). Chlorophyll *a* reacted most sensitively to stress. In ‘Lada’, the concentration of chlorophyll *a* decreased by more than 50% at 10 μM cadmium compared to the control (ANOVA_Cd_: *p* < 0.001; ANOVA_UV-B_: *p* > 0.05; ANOVA_Cd×UV-B_: *p* < 0.05). In ‘Ester’, chlorophyll *a* content was more stable at lower Cd concentrations, and a significant decrease occurred only at 20 μM Cd concentration (ANOVA_Cd_: *p* < 0.001; ANOVA_UV-B_: *p* > 0.05; ANOVA_Cd×UV-B_: *p* > 0.05). There was significant variability in the concentration of chlorophyll *b* due to cadmium stress in ‘Lada’ (ANOVA_Cd_: *p* < 0.001) and ‘Ester’ (ANOVA_Cd_: *p* < 0.05). In both ‘Lada’ and ‘Ester’, UV-B (ANOVA_UV-B_: *p* > 0.05) and the interaction between Cd and UV-B (ANOVA_Cd×UV-B_: *p* > 0.05) had no effect on chlorophyll *b* content. Carotenoids showed the same trend. In both cultivars, the content of carotenoids decreased only in response to cadmium stress (‘Lada’—ANOVA_Cd_: *p* < 0.001; ‘Ester’—ANOVA_Cd_: *p* < 0.001).

As an indicator of changes in the composition of the photosynthetic pigment antenna systems, we analysed the chlorophyll *a*/*b* ratio. It showed different patterns in the studied radish cultivars (Figure 4). In ‘Lada’, a slight decrease in the chlorophyll *a*/*b* ratio was recorded following the combined treatment with 10 µM Cd and UV-B irradiation. Both cadmium (ANOVA_Cd_: *p* < 0.05) and the interaction between cadmium and UV-B (ANOVA_Cd×UV-B_: *p* < 0.05) had a significant effect on the chlorophyll *a*/*b* ratio. In ‘Ester’, the chlorophyll *a*/*b* ratio was relatively stable, and significant changes occurred only in plants treated with 20 µM Cd in combination with UV-B. As in ‘Lada’, considerable changes in the chlorophyll *a*/*b* ratio occurred in response to cadmium treatment (ANOVA_Cd_: *p* < 0.05) and the interaction between the two stressors (ANOVA_Cd×UV-B_: *p* < 0.05). The relatively stable or slightly increasing trend in the chlorophyll *a*/*b* ratios suggests that, in both radish cultivars, cadmium affected chlorophyll *a* and chlorophyll *b* to a similar extent.

### 3.4. Specific Activity of SOD

**Figure 5:**
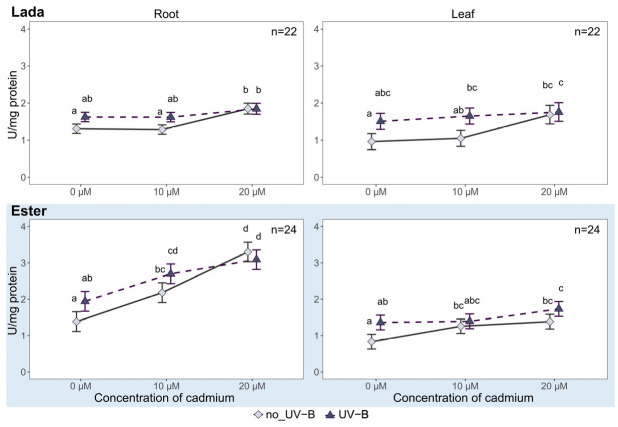
Effect of cadmium, UV-B and their interaction on the specific activity of SOD in roots and leaves of two radish cultivars—‘Lada’ and ‘Ester’. Data points represent means and error bars indicate confidence intervals (CIs); *n* indicates the sample size. Different letters indicate statistically significant differences between treatments (Tukey’s HSD test, *p* < 0.05).

Increasing cadmium concentration caused a significant increase in the specific activity of SOD (Figure 5). In both roots and leaves of ‘Lada’, the values of the specific SOD activity remained relatively stable. We recorded an increase in the activity of this enzyme only in plants treated with cadmium (ANOVA_Cd_: *p* < 0.001). A sharp increase was recorded in the roots of ‘Ester’, where the SOD activity at 20 µM Cd reached 3.36 U/mg of protein, representing more than a twofold increase compared to the control (1.40 U/mg of protein). The differences were caused by cadmium (ANOVA_Cd_: *p* < 0.001) and its interaction with UV-B (ANOVA_Cd×UV-B_: *p* < 0.05). In the leaves of this cultivar, the stress response was milder, with maximum values not exceeding 2.0 U/mg of protein. SOD activity was significantly stimulated by cadmium (ANOVA_Cd_: *p* < 0.001).

### 3.5. Specific Activity of CAT

**Figure 6:**
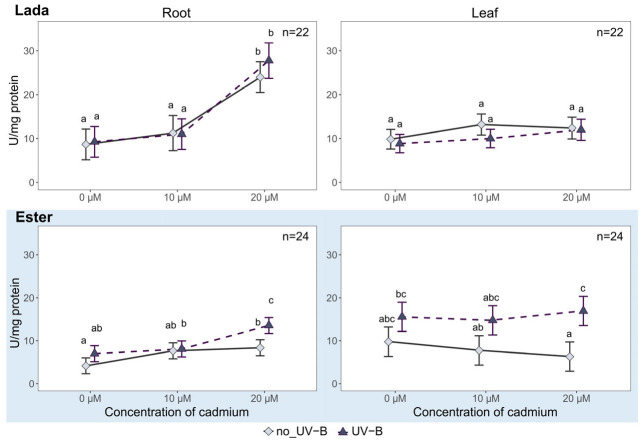
Effect of cadmium, UV-B and their interaction on the specific activity of CAT in roots and leaves of two radish cultivars—‘Lada’ and ‘Ester’. Data points represent means and error bars indicate confidence intervals (CIs); *n* indicates the sample size. Different letters indicate statistically significant differences between treatments (Tukey’s HSD test, *p* < 0.05).

Specific CAT activity showed different dynamics depending on the organ and treatment (Figure 6). In the roots of ‘Lada’, a sharp, threefold increase in CAT activity was recorded at 20 µM Cd (ANOVA_Cd_: *p* < 0.001), indicating substantial hydrogen peroxide accumulation in the root system. The interaction between the two stressors also had a statistically significant effect on the roots of this cultivar (ANOVA_Cd×UV-B_: *p* < 0.001). In the leaves of ‘Lada’, we observed no significant changes in the activity of this enzyme due to selected stressors (ANOVA_Cd_: *p* > 0.05; ANOVA_UV-B_: *p* > 0.05; ANOVA_Cd×UV-B_: *p* > 0.05).

‘Ester’ showed lower specific catalase activity in the roots compared to ‘Lada’. Root CAT activity was significantly affected by cadmium (ANOVA_Cd_: *p* < 0.001), UV-B radiation (ANOVA_UV-B_: *p* < 0.05) and the interaction between these stressors (ANOVA_Cd×UV-B_: *p* < 0.05). In leaf tissues, only UV-B radiation had a significant effect on CAT activity (ANOVA_UV-B_: *p* < 0.05).

### 3.6. Specific Activity of APX

**Figure 7:**
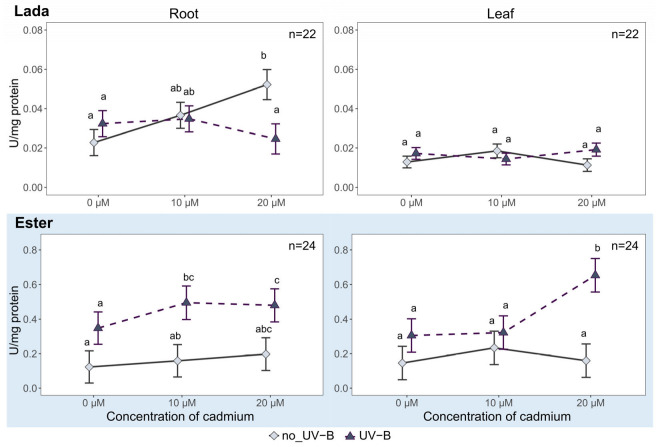
Effect of cadmium and UV-B radiation on the specific activity of APX in roots and leaves of two radish cultivars—‘Lada’ and ‘Ester’. Data points represent means and error bars indicate confidence intervals (CIs); *n* indicates the sample size. Different letters indicate statistically significant differences between treatments (Tukey’s HSD test, *p* < 0.05).

Analysis of specific APX activity revealed significant differences between the studied cultivars (Figure 7). The root system of ‘Lada’ showed an increasing trend in APX activity after cadmium treatment (ANOVA_Cd_: *p* < 0.05). In contrast, when cadmium and UV-B effects were combined, a significant decrease was observed (ANOVA_Cd×UV-B_: *p* < 0.05). Overall, APX activity in the leaves of ‘Lada’ was relatively stable, although statistically significant changes in activity of APX occurred under combined cadmium and UV-B stress (ANOVA_Cd×UV-B_: *p* < 0.05).

In ‘Ester’, the values of specific APX activity were up to ten times higher than those in ‘Lada’. In the roots of this cultivar, we noted a stimulating effect of cadmium (ANOVA_Cd_: *p* < 0.05) and UV-B irradiation (ANOVA_UV-B_: *p* < 0.05) when applied separately. However, the combination of these stressors did not have a significant effect on APX activity. In the leaves, the most pronounced stress response was recorded when UV-B treatment was combined with the highest concentration of cadmium (20 µM), where APX activity reached a maximum. Both stressors (ANOVA_Cd_: *p* < 0.001; ANOVA_UV-B_: *p* < 0.05) and their interaction (ANOVA_Cd×UV-B_: *p* < 0.001) had a significant impact on the specific activity of APX.

### 3.7. Specific Activity of GPOX

**Figure 8:**
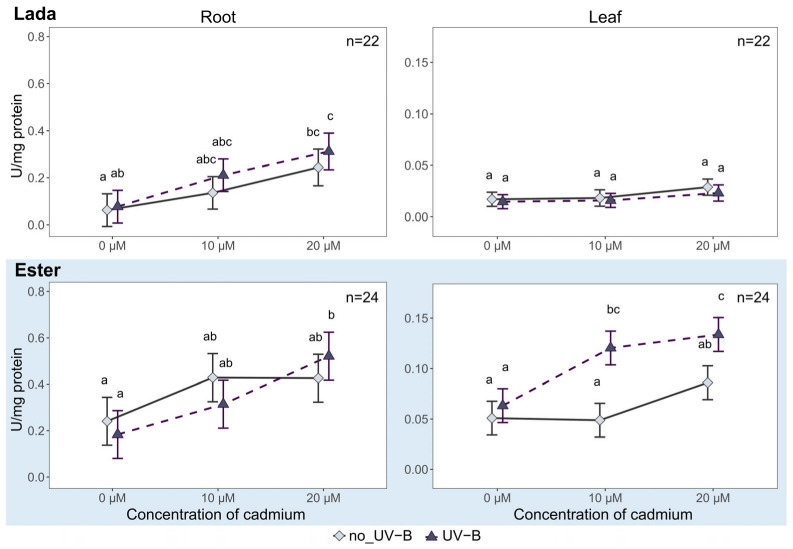
Effect of cadmium and UV-B radiation on the specific activity of GPOX in roots and leaves of two radish cultivars—‘Lada’ and ‘Ester’. Data points represent means and error bars indicate confidence intervals (CIs); *n* indicates the sample size. Different letters indicate statistically significant differences between treatments (Tukey’s HSD test, *p* < 0.05).

Analysis of specific GPOX activity showed significantly higher values in the root tissues compared to the leaves, reflecting the direct exposure of the root system to cadmium (Figure 8). In both cultivars, a gradual increase in GPOX activity was recorded in the roots with increasing cadmium concentration. In ‘Lada’ roots, only cadmium caused an increase in the activity of this enzyme (ANOVA_Cd_: *p* < 0.001).

Neither UV-B nor the interaction between Cd and UV-B had a significant effect. The leaves of ‘Lada’ showed only a minimal response of GPOX to the stress treatments (ANOVA_Cd_: *p* > 0.05; ANOVA_UV-B_: *p* > 0.05; ANOVA_Cd×UV-B_: *p* > 0.05).

The cultivar ‘Ester’ was generally characterized by higher GPOX activity than ‘Lada’. In the roots, GPOX activity increased gradually with increasing Cd concentration (ANOVA_Cd_: *p* < 0.001). UV-B had no significant effect on root GPOX activity. In contrast, in the leaves, cadmium (ANOVA_Cd_: *p* < 0.001) and UV-B (ANOVA_UV-B_: *p* < 0.05) caused a significant increase in GPOX activity. A synergistic effect of UV-B irradiation and cadmium was observed. While under treatments without UV-B, GPOX activity remained relatively stable, the combined treatment with UV-B and 20 µM Cd resulted in an almost threefold increase compared to the control (ANOVA_Cd×UV-B_: *p* < 0.05).

## 4. Discussion

### 4.1. Effect of Cadmium and UV-B on Production of Plant Biomass

Cadmium is a heavy metal that pollutes soil and water, negatively affecting plants. In addition, it poses a risk to both animals and humans because plants can accumulate cadmium and transfer it through the food chain [64]. As previously reported, the phytotoxic effects of cadmium lead to changes in plant metabolism by affecting mineral and water uptake and protein synthesis [65,66], resulting in a growth inhibition in plants. Both radish cultivars studied showed considerable sensitivity to cadmium exposure. Increasing cadmium concentration resulted in reduced biomass production, while the effect of UV-B on the fresh weight of the selected cultivars was not significant. ‘Lada’ exhibited a significant reduction in fresh weight at just 10 μM cadmium, while in ‘Ester’ growth inhibition at this concentration was less pronounced.

### 4.2. Variability of Endopolyploidy Induced by Cadmium Stress, While UV-B Had No Effect

Cadmium also causes changes in the synthesis and signalling of phytohormones. Recent studies indicate that auxin acts as a central regulator of abiotic stress responses through extensive crosstalk with cytokinin [67] and other phytohormones, coordinating cell proliferation, differentiation, and endoreduplication [68]. Cadmium-induced disruption of auxin transport [69] and cytokinin signalling may suppress cyclin/CDK activity and meristematic cell proliferation [70]. At the same time, cadmium-generated oxidative stress and DNA damage can activate cell-cycle checkpoint pathways, including SOG1- and WEE1-mediated signalling [71,72], thereby inhibiting mitosis and promoting repeated rounds of DNA replication without cell division. Thus, rather than the AUX:CK balance alone, the combined effects of altered phytohormone homeostasis [73], oxidative stress [15], and DNA-damage signalling [74] may redirect cells from mitotic cycles to endoreduplication, allowing continued metabolic activity under stress. However, since phytohormone levels and cell-cycle regulators were not analysed in the present study, this interpretation should be regarded as a plausible mechanistic hypothesis rather than a conclusion directly supported by our experimental data.

Cadmium has been reported to either stimulate or inhibit endopolyploidy depending on the species or plant organs. Previous studies reported increased endopolyploidy in roots [45,46] and conversely reduced endopolyploidy levels in leaves [75]. In storage hypocotyls of ‘Ester’, cadmium exposure appeared to suppress endoreduplication. In storage hypocotyls of ‘Lada’, endopolyploidy decreased slightly, but it seemed that combined treatment of 20 μM Cd and UV-B resulted in a slight increase in EI, at the borderline of statistical significance. Previous reports suggest that moderate UV-B exposure may promote endoreduplication [42,76], which could partially offset cadmium-induced effects. Nevertheless, the contribution of UV-B to endopolyploidy maintenance remains to be clarified.

We observed stagnation in endopolyploidy in leaves and petioles. Since cadmium accumulates predominantly in roots rather than in shoots [77], the lower exposure of these organs to cadmium may have contributed to the absence of changes in their endoreduplication index (EI). However, cadmium accumulation was not quantified in the present study; therefore, this explanation remains hypothetical. Our study confirmed the organ-specificity of endopolyploidy [2,14], with the highest endopolyploidy in petioles and the lowest in leaves of both cultivars.

### 4.3. Cadmium and UV-B Negatively Impact Concentration of Assimilation Pigments

The content of assimilatory pigments decreased with increasing cadmium concentration. We noted a decreasing trend in the content of assimilatory pigments with increasing cadmium concentration as previously observed [64], with the most significant decrease in the content of chlorophyll *a*. Cadmium-induced reductions in chlorophyll content have been attributed to inhibition of chlorophyll biosynthesis [78], oxidative degradation of photosynthetic pigments [79], and displacement of Mg^2+^ from the chlorophyll porphyrin ring [80], leading to destabilization of chlorophyll molecules. Because chlorophyll *a* is predominantly associated with the reaction centres of photosystems I and II, whereas chlorophyll *b* is mainly localized in the light-harvesting complexes, cadmium stress often results in a greater decline in chlorophyll *a* than chlorophyll *b*, reflecting preferential damage to the photosynthetic reaction centres [52,81]. Consequently, changes in the chlorophyll *a*/*b* ratio are commonly associated with alterations in the photosynthetic apparatus and reduced photosynthetic efficiency under heavy metal stress [52]. Changes in the chlorophyll *a*/*b* ratio differed between the two cultivars under stress conditions, indicating cultivar-specific responses of the photosynthetic apparatus. In ‘Lada’, the chlorophyll *a*/*b* ratio decreased in plants treated with UV-B under the influence of 10 μM then increased again at 20 μM cadmium treatment. In contrast, the chlorophyll *a*/*b* ratio in ‘Ester’ remained relatively stable across most treatments but declined under combined treatment of 20 μM and UV-B.

### 4.4. Antioxidant Enzyme Response to Oxidative Stress Caused by Cadmium and UV-B

The results of the analysis of the antioxidant activities of selected enzymes indicate that both ‘Lada’ and ‘Ester’ plants indeed faced significant oxidative stress. The first line of defence was provided by SOD, which detoxifies O_2_^−^, resulting in the formation of H_2_O_2_ [82,83]. SOD activity in both roots and leaves of ‘Lada’ and ‘Ester’ increased proportionally with increasing cadmium concentration, with UV-B irradiation further enhancing this response. The strongest increase in SOD activity was observed in the roots of ‘Ester’, suggesting enhanced accumulation of O_2_^−^ radicals in this organ.

CAT, APX, and GPOX also responded to the increased concentration of H_2_O_2_ in tissues, resulting from detoxification of ROS. CAT is characterized by its high efficiency and is considered a metabolically less demanding pathway for H_2_O_2_ detoxification than the ascorbate–glutathione cycle [84]. ‘Ester’ showed a slight increase in CAT activity mainly due to UV-B, whereas ‘Lada’ showed a sharp increase in CAT activity, especially in the roots. The preferential activation of CAT in ‘Lada’ coincided with a greater maintenance of endopolyploidy under stress. Although this association suggests that antioxidant strategy may influence cellular responses to cadmium, our data do not allow us to establish a direct causal relationship between CAT activity and endopolyploidy maintenance. Similar cultivar-specific differences in antioxidant enzyme activation have been reported in plants exposed to heavy metal stress, reflecting different physiological strategies for coping with oxidative damage [81].

The results also revealed fundamental differences in APX activity. APX, like CAT, participates in the degradation of ROS, through the ascorbate–glutathione cycle [56]. In the present study, ‘Ester’ exhibited up to tenfold higher APX activity than ‘Lada’, indicating a greater reliance on this antioxidant pathway. However, APX activation requires a more energy-intensive supply of ascorbate and NADPH [85], which represents a significant metabolic burden. The stronger activation of APX in ‘Ester’ may reflect a greater metabolic investment in antioxidant defence, which could potentially influence other energy-demanding cellular processes, including endoreduplication. However, this interpretation remains hypothetical because metabolic allocation and cell-cycle regulation were not directly investigated. APX activity was even higher in leaves, since this enzyme partially compensates for damage to the photosynthetic apparatus under the influence of UV-B radiation [85]. In ‘Lada’, APX activity remained relatively stable, with more pronounced activation observed mainly in roots. These cultivar-specific differences suggest distinct antioxidant response strategies under combined cadmium and UV-B stress.

Because UV-B primarily affects above-ground tissues, its effect on GPOX activity was even more pronounced in leaves than in roots. UV-B radiation increased the activity of GPOX in the roots only in combination with 20 μM Cd. In leaves, ‘Ester’ exhibited a marked increase in GPOX activity, which was further enhanced by UV-B treatment, whereas ‘Lada’ showed relatively stable GPOX activity regardless of UV-B exposure. Similar increases in GPOX activity under heavy metal stress have been associated with reinforcement of the cell wall through lignification [86]. In the roots, the higher GPOX activity could therefore be related to the attempt to prevent apoplastic transport of cadmium deeper into the tissues, although this mechanism was not directly examined in the present study.

### 4.5. Modulatory Effect of UV-B on Cadmium-Induced Toxicity

Previous studies show various negative effects of UV-B on plants, including defective growth, reduction in biomass, damage of photosystems [42] and DNA damage [43], resulting in activation of enzymatic antioxidants and an increase in endopolyploidization [15,76]. In general, it seems that cadmium caused stronger oxidative stress to studied radish cultivars than UV-B. UV-B alone affected only the activity of CAT, APX and GPOX in the ‘Ester’ cultivar. Although the UV-B treatment applied in the present study did not induce severe stress, it significantly modified the response of plants to cadmium, particularly through changes in antioxidant enzyme activities. One possible explanation is the activation of the UVR8-mediated signalling pathway, which regulates the expression of antioxidant genes and flavonoid biosynthesis in response to UV-B exposure [87]. Such responses may prime plants for subsequent abiotic stress and thereby modulate cadmium-induced oxidative responses [88]. Since UVR8 signalling and flavonoid accumulation were not investigated in the present study, this mechanism should be regarded as a plausible hypothesis based on previous studies rather than a conclusion supported by our experimental data.

### 4.6. Cultivar-Specific Stress Response Strategies and Trade-Offs Between Antioxidant Activity and Overall Fitness

The ‘Lada’ and ‘Ester’ cultivars differed significantly in their stress response strategies. ‘Lada’ appeared to rely preferentially on CAT-mediated detoxification, which is generally considered a more energy-efficient antioxidant pathway [89] and was associated in the present study with greater maintenance of endopolyploidy under stress. In contrast, ‘Ester’ showed stronger activation of APX- and GPOX-related antioxidant responses, suggesting a greater reliance on metabolically demanding defence strategy [89], which in the present study coincided with reduced endopolyploidy in storage hypocotyls. Our results indicate that the two cultivars adopted distinct antioxidant response strategies under combined cadmium and UV-B stress. The maintenance of endopolyploidy in ‘Lada’ was associated with a different pattern of antioxidant enzyme activation from that observed in ‘Ester’. Whether these contrasting antioxidant strategies directly influence endopolyploidy maintenance remains to be determined.

### 4.7. Study Limitations and Future Perspectives

Although the present study provides new insights into the relationship between endopolyploidy and antioxidant responses under combined cadmium and UV-B stress, several limitations should be acknowledged. We focused primarily on endopolyploidy, biomass production, photosynthetic pigment content, and antioxidant enzyme activities. Additional physiological and biochemical analyses, including chlorophyll fluorescence, gas exchange measurements, reactive oxygen species (ROS) and hydrogen peroxide quantification, lipid peroxidation, and membrane stability assessed by electrolyte leakage, would provide a more comprehensive understanding of the mechanisms underlying stress tolerance. Furthermore, quantification of cadmium accumulation in individual organs together with phytohormone profiling would help clarify the molecular basis of the cultivar-specific responses observed in this study. Although beyond the scope of the present study, emerging cell-type-resolved approaches, including single-cell transcriptomics, offer new opportunities to dissect the spatial and molecular heterogeneity of plant stress responses and may provide further insight into the regulation of endopolyploidy under combined abiotic stresses [90].

## 5. Conclusions

The present study showed that cadmium-induced oxidative stress strongly affected the physiological status of both *Raphanus sativus* L. cultivars, whereas UV-B radiation alone had only a limited effect. Nevertheless, UV-B modulated the plant response to cadmium, particularly through changes in antioxidant enzyme activities and chlorophyll balance. The two cultivars exhibited distinct stress-response strategies. ‘Lada’ maintained relatively stable endopolyploidy levels and preferentially activated CAT-mediated detoxification, which is generally considered a more energy-efficient antioxidant strategy. In contrast, ‘Ester’ displayed stronger activation of APX- and GPOX-dependent antioxidant pathways, accompanied by a reduction in endopolyploidization in storage hypocotyls. These findings suggest that maintenance of endopolyploidy was associated with greater tolerance to combined cadmium and UV-B stress in the cultivar ‘Lada’. However, whether endopolyploidy directly contributes to stress tolerance or represents a correlated response requires further investigation.

## Data Availability

The raw data supporting the conclusions of this article will be made available by the authors on request.

## Supplementary Materials

The following supporting information can be downloaded at: https://www.mdpi.com/article/10.3390/plants15152320/s1. Figure S1: Representative photographs of plants after 50 days of cultivation showing the visible effects of UV-B irradiation and CdCl_2_ treatment. UV-B-treated plants exposed to higher Cd concentrations exhibited pronounced chlorosis, necrosis, leaf senescence, and growth inhibition, confirming the severity of the applied stress conditions. Bar = 10 cm.
