# Supplementary material for: Abiotic Stress Responses in Raphanus sativus L.: Insights into the Role of Endopolyploidization in Stress Adaptation
Source: Plants (Basel). 2026 Jul 28;15(15):2320. doi: 10.3390/plants15152320 (PMC13468157; doi:10.3390/plants15152320)

Supplementary Figure S1. Representative photographs of plants after 50 days of cultivation showing the visible effects of UV-B irradiation and $CdCl_2$ treatment. UV-B-treated plants exposed to higher Cd concentrations exhibited pronounced chlorosis, necrosis, leaf senescence, and growth inhibition, confirming the severity of the applied stress conditions. Bar = 10cm

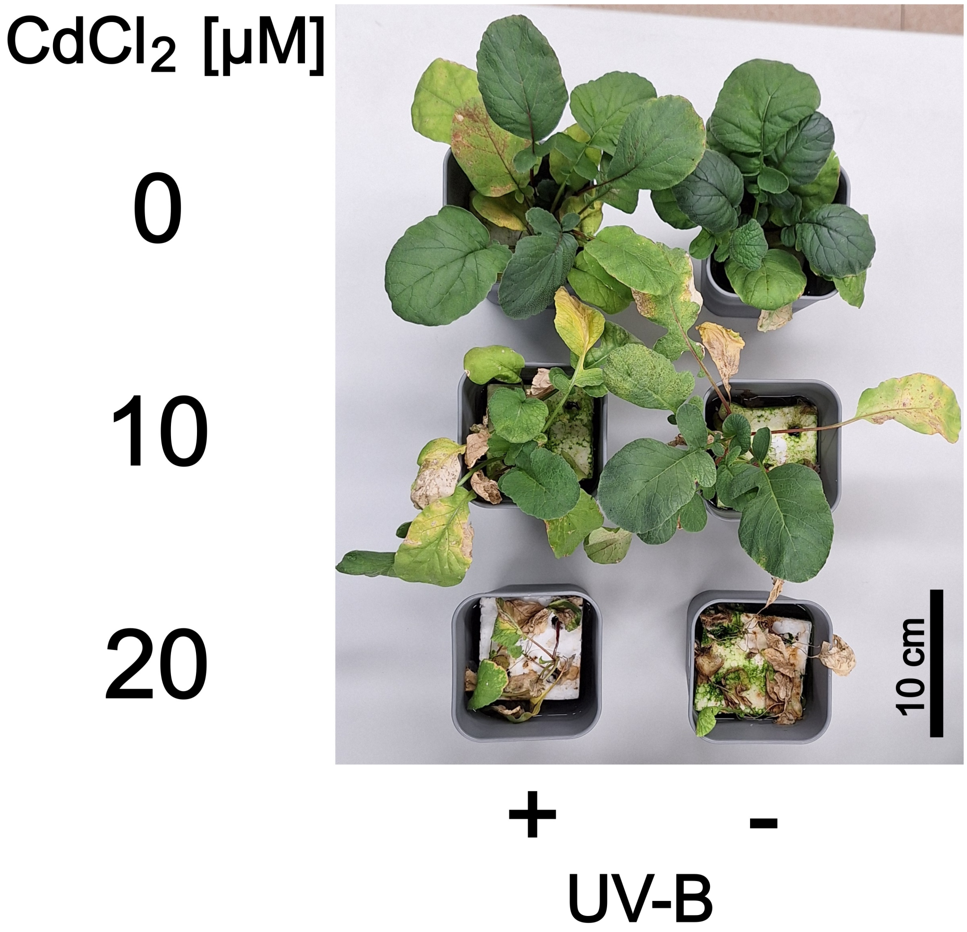

Supplement: Supplementary file 1 [file plants-15-02320-s001.zip › plants-4413442-supplementary.pdf]
